# Ferroelectric Phase Transition in Barium Titanate Revisited with Ab Initio Molecular Dynamics

**DOI:** 10.3390/ma17051023

**Published:** 2024-02-23

**Authors:** Christian Ludt, Dirk C. Meyer, Matthias Zschornak

**Affiliations:** 1 Institute of Experimental Physics, Technische Universität Bergakademie Freiberg, 09599 Freiberg, Germany; dirk-carl.meyer@physik.tu-freiberg.de (D.C.M.); matthias.zschornak@physik.tu-freiberg.de (M.Z.); 2 Zentrum für Effiziente Hochtemperatur-Stoffwandlung, 09599 Freiberg, Germany; 3 Technical Physics, Hochschule für Technik und Wirtschaft Dresden, 01069 Dresden, Germany

**Keywords:** ferroelectric phase transition, barium titanate, ab initio molecular dynamics

## Abstract

The ferroelectric phase transition of the perovskite barium titanate as well as its technical importance regarding the switching of respective polar properties is well known and has been thoroughly studied, both experimentally and on theoretical grounds. While details about the phase diagram as well as transition temperatures are experimentally well known, the theoretical approaches still face difficulties in contributing a detailed description of these phase transitions. Within this work, a new methodological approach is introduced to revisit the ferroelectric phase transition with first-principles methods. With the chosen ab initio molecular dynamics (AIMD) method in combination with the applied NpT ensemble, we are able to join the accuracy of density functional theory (DFT) with ambient conditions, realized using a thermostat and barostat in an MD simulation. The derived phase diagram confirms recent corrections in the theoretical models and reproduces the phase boundary pressure dependence of *T*_C_. In conclusion of the statistical atomistic dynamics, the nature of the transition can be described in a more detailed way. In addition, this work paves the way towards locally patterned piezoelectrica by means of acoustic standing waves as well as piezoelectrically induced acoustic resonators.

## 1. Introduction

Ferroelectric materials, as well as their phase transitions, are important phenomena for technical applications [1,2]. They exhibit an electric dipole within the unit cell. The occurring spontaneous polarization can be controlled through external perturbations like temperature or pressure as well as be reoriented using electric fields [3]. At a given temperature, the so-called *Curie temperature* (*T*_C_), the unit cell undergoes a structural change to a centrosymmetric cell, loses its polarization, and becomes paraelectric.

Barium titanate (BaTiO_3_) is the prototype representative of ferroelectric perovskites. With properties like piezoelectricity, dielectric susceptibility, good optical and transport attributes, multiferroism, etc. [4,5], it is an interesting functional material due to its variety of possible applications, e.g., nonvolatile memory, high-κ dielectrics, and piezoelectric sensors [6,7,8,9,10].

Barium titanate appears in a variety of phases: It undergoes phase transitions from a cubic to tetragonal crystal structure at 393 K, tetragonal to orthorhombic at 278 K, and orthorhombic to rhombohedral at 183 K [11]. The phase transition between the ferroelectric tetragonal phase (*P*4*mm*) and the paraelectric cubic phase (*Pm*$\bar{3}$*m*) is of great interest for technical applications. It arises in competition with long-ranged ordered and short-ranged ordered forces, breaking crystal symmetry by displacing the B-cation or tilting the O-octahedra [4,12,13]. In the literature, two mechanisms are discussed governing this transition: the order–disorder mechanism [8,14,15] or the displacive mechanism [16,17,18]. There are experimental indications that the displacive model is more realistic far away from *T*_C_, while the picture of order–disorder is more appropriate in the vicinity of the transition [19,20].

The focus of this paper is to revisit this phase transition, driven by temperature as well as external pressure, in a theoretical approach, in order to reveal the influence of external strain effects. This will be useful to improve experimental settings, in which *standing acoustic waves* are used to control crystal properties locally. A description of this method is found in previous work [21,22]. Here, piezoelectric resonators are used to couple standing acoustic waves into crystals to change the symmetry locally and to introduce a patterning of the phase distribution within a given crystal. In previous work, we were also able to present the effect of internal strain on strontium titanate, like that it is induced by stacking faults [23,24] or in response to an applied electric field [25,26,27]. With our work, we want to show new paths of controlling crystals’ properties through internal and external effects, which may lead to new promising applications.

The nature of the ferroelectric phase transition has been analyzed experimentally as well as theoretically [28]. While the transition is experimentally well described [29,30] and semi-empiric approaches like force-field MD (FF-MD) are well developed [31,32,33], ab initio methods still have problems in predicting the including processes, in particular, thermal expansion, correctly. While the mentioned FF-MD methods are able to handle supercells of 10 × 10 × 10 and larger for long simulation times of hundreds of pico seconds, the empirically chosen force-field contains the disadvantage that it does not have the predictive power and electronic degrees of freedom of the ab initio level. The ab initio molecular dynamics (AIMD) methods introduce technical-based limits to the supercell size, but the more detailed description of interatomic forces is a big advantage and will enhance the flexibility in novel materials research. As is pointed out in the literature [19], inaccuracies in the description of the interplay between short-ranged repulsive forces and the long-ranged coulomb interactions lead to large errors in the ferroelectric well depths vs. volume and this affects especially sensitive quantities like the *Curie temperature T*_C_. In the literature, an NpT-AIMD study is presented by Qi et al. [33]. Even though their state-of-the-art simulations comprise larger supercells and simulation times, they struggle to predict the experimental value of *T*_C_. In addition, in their work the phase transition is not analyzed under the influence of external pressure. In our work, with this additional scope, we aim to present the pT-phase diagram on an AIMD basis.

In this paper, ab initio molecular dynamics [34] is used to model the phase transition of barium titanate. By these means, forces determined via *density functional theory* (DFT) are utilized in the *molecular dynamics* (MD) to simulate the trajectory of the given system. The isothermal–isobaric (NpT) ensemble is chosen, which allows for a dynamic volume and therefore additional degrees of freedom, i.e., varying lattice parameters. The velocity of the system’s particles is controlled by a thermostat, which governs the average kinetic energy in the system, i.e., the temperature, as well as a barostat, which implies an external pressure on the system. With this combination of methods, we are able to analyze the ensemble in more realistic conditions, compared to the DFT ground state on one hand or an empirically established MD on the other. The aim is to develop a precise atomistic model that is able to simulate the phase transition controlled by the combined intensive thermodynamic state variables temperature and pressure in order to gain a better insight into the atomistic structural response and related phenomena for the prototype ferroelectric perovskite barium titanate. While a lot of MD simulations and DFT calculations are found in the literature, a combination of both is quite scarce, and the chosen (NpT) ensemble is rarely applied in the literature yet.

## 2. Methods

The DFT calculations were performed with the *Vienna Ab initio Simulation- Package* [35,36,37] (VASP). The potentials were treated within the projector augmented wave method [38] (PAW), while the exchange–correlation functional was approached in *generalized gradient approximation* (GGA) using the PBEsol functional [39], combined with Hubbard U corrections for the Ti *d*-states, which were chosen in dependence of the supercell size, as explained later. For the AIMD calculations within the framework of an NpT ensemble, the *Langevin* thermostat [40,41,42] was used to control temperature and a *Parinello–Rahman* barostat [43,44] to control the external pressure.

The requirements on accuracy have been tested in several series of calculations, with varied cut-off energies up to 600 eV, resulting in the setting of 450 eV, which converges the total energy within 10^−4^ eV. The sampling in the reciprocal space was realized using a gamma-only setting. AIMD calculations were performed at selected temperatures and external pressures with an MD step size of 1 fs for 6000 steps, i.e., a 6 ps time span in total.

Additionally, 4 × 4 × 4 supercells have been calculated, in order to check the convergence with the system size. The time period was reduced to 5000 steps of 1 fs, i.e., 5 ps, because of the high computational costs of these calculations.

## 3. Results

### 3.1. Polarization Switching Due to Temperature

First, the tetragonal phase of barium titanate was modeled as an initial ensemble for the AIMD calculations. The unit cell was expanded to supercells of different dimensions, which were tested at chosen temperatures realized using the *Langevin* thermostat in order to find changes of the initial polarity. Hubbard U was chosen to be 4 eV on Ti *d*-states according to the literature [45].

**Figure 1:**
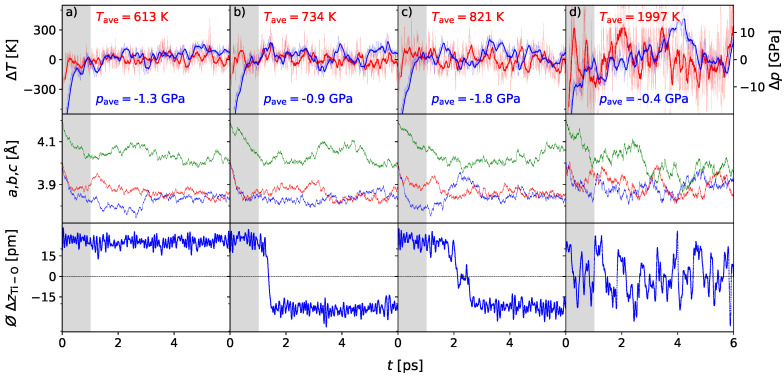
AIMD simulation of the BaTiO_3_ 2 × 2 × 2 supercell at 600 (**a**), 700 (**b**), 800 (**c**), and 2000 K (**d**) and 0 GPa external pressure: The area of the first picosecond is highlighted gray to mark the relaxation time, which is needed to approach the thermodynamic equilibrium. Lattice parameters *a, b,* and *c* given in red, blue and green, indicate the tetragonal phase in the cases of 600, 700, and 800 K. At 2000 K, the lattice parameters approach each other, indicating a cubic phase. The mean displacements of titanium ions in comparison to the respective oxygen planes in *z*-direction $⌀\Delta z_{\text{Ti-O}}$ show different behavior depending on temperature: In case of the 600 K simulation, $⌀\Delta z_{\text{Ti-O}}$ is stable at approximately 23 pm, reflecting that the phase is polar, while at 700 K a polarization switching occurs, which is given by the changed sign of $⌀\Delta z_{\text{Ti-O}}$. At 800 K, the behavior is alike, but for a short time a centrosymmetric plateau can be established. In the case of 2000 K, the displacement parameter $⌀\Delta z_{\text{Ti-O}}$ switches frequently.

In Figure 1, important simulated parameters are shown for the 2 × 2 × 2 supercell at different temperatures, i.e., 600, 700, 800, and 2000 K. For each simulation, the temperature and pressure are plotted for every step within the 6 ps of simulation time. Also given are the lattice parameters for the unit cells. The bottom plot presents the mean value of all *z*-distances between the Ti-ions and the respective oxygen planes $⌀\Delta z_{\text{Ti-O}}$. While this parameter is an important quantity to give a prediction to the polarity and hence the ferroelectricity of the supercell under the chosen conditions, the respective quantities $⌀\Delta x_{\text{Ti-O}}$ and $⌀\Delta y_{\text{Ti-O}}$ are equal to zero and therefore have no influence on the total polarity. The gray area from 0 to 1000 fs marks the relaxation time until thermodynamic equilibrium is reached. Here, the system strongly fluctuates due to the contact and energy transfer with the thermostat and barostat. Data within this area provide details about parameter convergence and relaxation within the selected time regime.

Taking a closer look at the graphs of temperatures and pressures, oscillations of about ΔT/T=10% and Δp=±50 kBar occur in both quantities around the desired values, while the system keeps the desired average temperature and pressure within the chosen simulation time of 6 ps. The system at 600 K is presented in Figure 1a. Here, the lattice parameters also adapt to the changed external conditions. With small fluctuations of less than 0.2 Å, no qualitative change appears. The c/a ratio still indicates the tetragonal phase. The mean displacement of the Ti-ions to their respective O-plane is stable at about $\Delta z_{\text{Ti-O}}$ = 25 pm, showing no variation. The initial polarity of the tetragonal phase is maintained for this set of external conditions. For the system at 700 K presented in Figure 1b, the development of the temperature and pressure with time is comparable to the dynamics of the ensemble at 600 K, and the lattice parameters indicate a tetragonal phase, as before. However, a remarkable polarization switching occurs, which is evident from the displacement of the Ti-ions between displacements $\Delta z_{\text{Ti-O}}$ = ±23 pm. The temperature-induced kinetic energy of the Ti-ions is apparently high enough to overcome the energetic barrier, which represents the tunneling of the Ti ions through the oxygen plane. The behavior at higher temperatures is shown in Figure 1c, exemplarily for T = 800 K. Here, the lattice parameters show a new feature. The c/a ratio changes dramatically within the first few picoseconds with temporary values closer to the cubic phase and then stabilizes, as seen before. At the same time, the displacement shows a switching between both directions of polarization, with a short non-polar plateau for several 100 femtoseconds in between. This is interpreted as a first indication of an order–disorder stabilizing the average centrosymmetric phase. Finally, at 2000 K (Figure 1d), the features emphasized above stabilize during the MD calculation. The lattice parameters approach each other, indicating the cubic phase. The polarization states are stable only within small time intervals not exceeding 500 fs, with transient times of a few 10 fs, which is interpreted as centrosymmetry.

Summarizing the results of the 2 × 2 × 2 temperature calculations, a stable tetragonal phase is found at temperatures below 700 K. At this point, first polarization switching of the mean displacement occurs. On a time average >1 ps, these switching events destabilize the tetragonal structure and initiate the phase transition of the ensemble into the cubic phase. The representative switching frequency increases to approximately 1/100 fs at a temperature of 2000 K, which further stabilizes the cubic phase.

**Figure 2:**
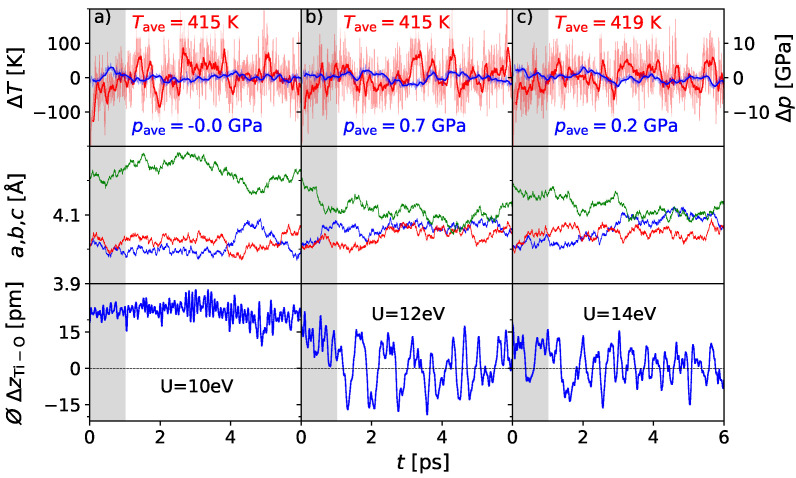
AIMD simulation of BaTiO_3_ 2 × 2 × 2 supercells at 400 K for different Hubbard U values (10 eV (**a**), 12 eV (**b**) and 14 eV (**c**)). While the simulation at 10 eV shows tetragonal behavior over the whole simulation time, regarding lattice parameters (*a* in red, *b* in blue, *c* in green) as well as the displacements, cubic behavior is indicated at 12 eV and 14 eV.

While the qualitative behavior, i.e., the ferroelectric phase transition appearing at elevated temperatures, fits the expectations, the experimental *Curie temperature* of 393 K is not reproduced correctly. By scanning different influences on the energy landscape of barium titanate, the Hubbard U parameter was shown to be crucial to the transition temperature (Supplementary Section S3). In this way, the *Curie temperature* could be correctly reproduced only for a rather large value of 12 eV on the Ti *d*-sates in the case of 2 × 2 × 2 supercells. In Figure 2, the simulations are shown for 400 K and different Hubbard U values. It is evident that higher U values stabilize the cubic phase, which is consistent with the results from previous work [19]. The value of the Hubbard U parameter, which is needed to match the experimental Curie temperature, decreases with larger supercell sizes, which can be seen later. It can be concluded that the Hubbard U parameter seems to counteract effects that are introduced by a small supercell size.

**Figure 3:**
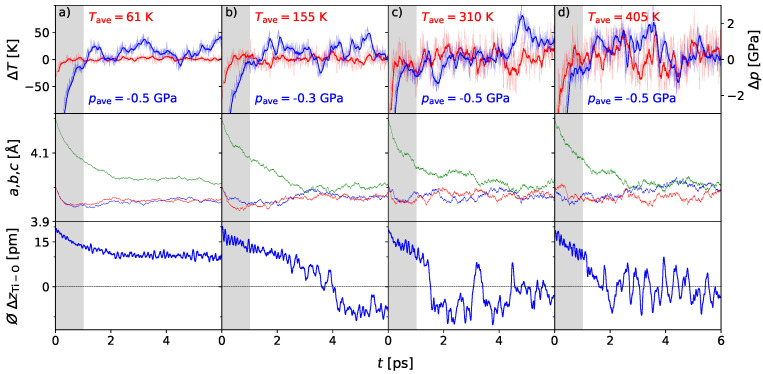
AIMD simulation of the BaTiO_3_ 3 × 3 × 3 supercell at 50 (**a**), 150 (**b**), 300 (**c**), and 400 K (**d**) and 0 GPa external pressure: The noise in the monitored quantities is reduced significantly compared to the 2 × 2 × 2 supercell. Lattice parameters *a*, *b*, and *c* given in red, blue and green, indicate the tetragonal phase for 50 and 150 K, while at 150 K, short cubic-like behavior appears. For the elevated temperatures of 300 and 400 K, the approaching lattice parameters indicate a predominantly cubic unit cell. The mean displacement $⌀\Delta z_{\text{Ti-O}}$ reflects a polar phase at 50 K. Switching begins to be established at 150 K and is more frequent as the temperature increases up to 400 K. Here, a rather constant rapid switching indicates the first appearance of a centrosymmetric phase.

Because of the high relative fluctuation of the external conditions, the temperature and pressure (ΔT/T=10% and Δp=±50 kBar), a larger supercell of 3 × 3 × 3 has been analyzed in the same way to increase the ensemble size and thus decrease respective variances. The results with a chosen Hubbard U parameter of 4 eV on the Ti *d*-states are presented in Figure 3.

The fluctuations in temperature and pressure drop significantly compared to the 2 × 2 × 2 supercell to confidence regions of ΔT/T<5% and Δp=±10 kBar. While at 50 K (Figure 3a) a stable polarization occurs over the full time regime of 6 ps, at 150 K the first switching of polarity in the *c*-direction can be identified. This switching appears more rapidly, with higher temperatures. At 400 K, the mean displacement shows again the frequent switching events are approximately each 200 fs, equivalent to the 2 × 2 × 2 supercell ensemble at 2000 K. Also, the three lattice parameters approach each other at ∼4 Å. Both indicate a phase transition from the polar tetragonal phase to the unpolar cubic phase. The temperature of ∼400 K matches with the Curie temperature reported in [46], which indicates that the size of the ensemble is sufficiently large to diminish boundary effects.

We also tested unsymmetric supercells. A discussion of the results on 2 × 2 × 4 and 4 × 2 × 2 supercells is given in the Supplementary Section S1.

**Figure 4:**
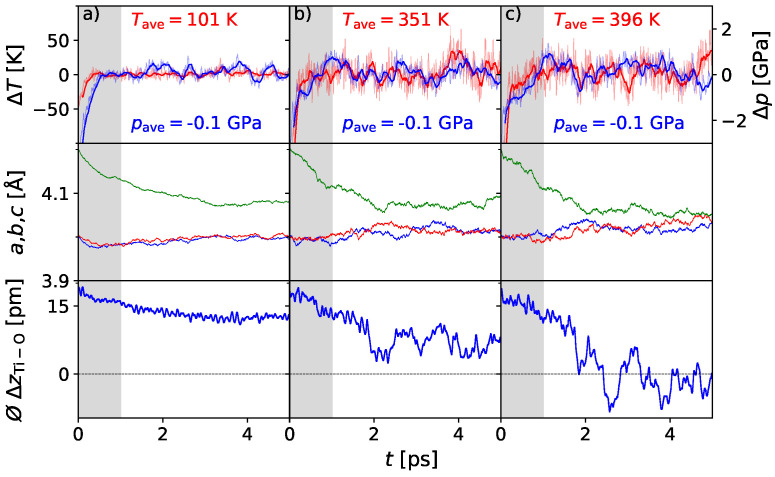
AIMD simulation of the BaTiO_3_ 4 × 4 × 4 supercell at 100 (**a**), 350 (**b**), and 400 K (**c**) and 0 GPa external pressure: Lattice parameters *a, b, c* given in red, blue and green, indicate the tetragonal phase for 100 and 350 K. For the elevated temperature of 400 K, the approaching lattice parameters indicate a cubic unit cell. The mean displacement $⌀\Delta z_{\text{Ti-O}}$ reflects a polar phase at 100 K and 350 K. At 400 K, a rather constant rapid switching indicates the first appearance of a centrosymmetric phase.

In addition, 4 × 4 × 4 supercells have also been modeled, and it was found that the energetical difference between the tetragonal polar phase and the cubic unpolar phase is strongly correlated to the chosen Hubbard U parameter. This dependence is known from the literature [47]. In order to reproduce the experimental *Curie temperature*, the Hubbard U parameter was chosen to be 2 eV on the titanium *d*-states. A series of different temperatures is presented in Figure 4. It can be seen that the phase transition again occurs between 350 K and 400 K. The convergence can be verified, even if a longer simulation time would be more beneficial in some cases. Compared to the work of Qi et al. [33], we are able to reproduce the ferroelectric phase transition with a smaller computational effort. The agreement of our calculations has been further improved using the Hubbard U parameter. Even though this parameter was set empirically for the computational series of the respective supercell, the method is convincing with correct statements on *T*_C_ and enables a description of phase transitions on an ab initio basis with reasonable effort.

### 3.2. Influence of Pressure on the Phase Transition

In a next step, we analyze how pressure affects the atomic arrangements, lattice parameters, and polarization states inside our supercells. With the use of a Parinello–Rahman barostat, external pressure was applied. We aim to extend the reported phase diagrams on the one hand and assist in investigating experimental settings of piezoelectrically induced acoustic resonators as described and used, e.g., by Eliovich et al. [21,22], Blagov et al. [48], and Marchenkov et al. [49], on the other hand.

Again, we studied the structural dynamics of the phase transition in dependence of the external pressure for the smaller ensemble as well as the larger ensemble within the 2 × 2 × 2 supercells, 3 × 3 × 3 supercells, and 4 × 4 × 4 supercells. Here, we present the results for the larger ensemble (3 × 3 × 3) with reduced variances in T and p; data for the smaller ensemble can be found in the Supplementary Section S2. As for the zero-pressure cases, we identified the characteristics of constant polarization, as well as beginning and frequent polarization switching.

**Figure 5:**
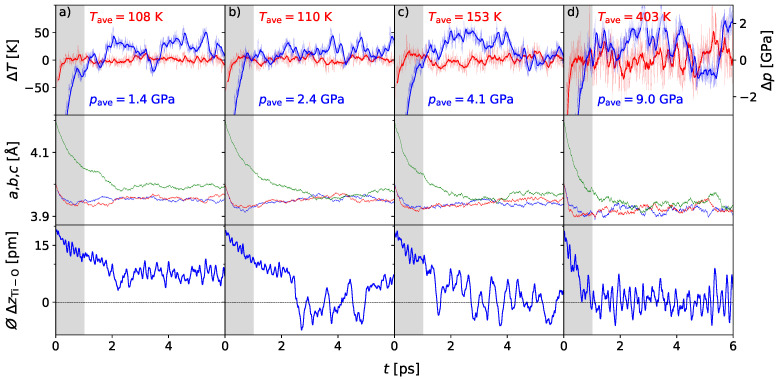
AIMD simulation of the BaTiO_3_ 3 × 3 × 3 supercell under the influence of chosen temperatures and external pressures: in (**a**) at 100 K and 2 GPa, (**b**) at 100 K and 3 GPa, (**c**) at 150 K and 5 GPa, (**d**) at 400 K at 10 GPa. Lattice parameters *a, b,* and *c* given in red, blue and green, indicate the cubic phase for all conditions. The mean displacement $⌀\Delta z_{\text{Ti-O}}$ reflects polar character for 100 K and 2 GPa, while for 100 K at 3 GPa the behavior represents a semi-state. For the other conditions, centrosymmetry is observed.

**Figure 6:**
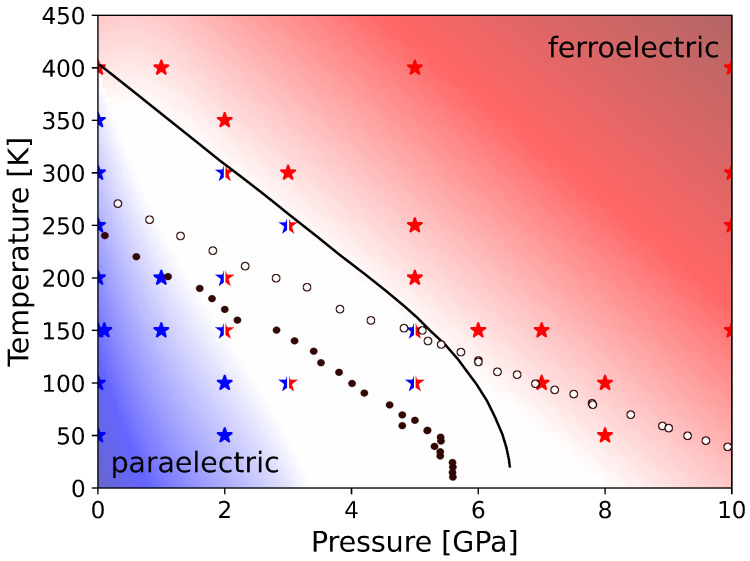
Phase diagram of BaTiO_3_. The colored stars display the AIMD results for ferroelectric phase in blue and paraelectric phase in red, respectively. Mixed stars represent MD calculations, which show a transition state. The solid line indicates the tetragonal to cubic phase transition proposed experimentally by Ishidate et al. [11]. The circles represent theoretical calculations by Íniguez and Vanderbilt [50], while solid circles give classical Monte Carlo simulations, and open circles are generated using the path-integral quantum Monte Carlo (PI-QMC) technique. Colored backgrounds emphasize the respective regions in the phase diagram based on our calculated data.

Exemplarily, we show the dynamics for the temperature level of 150 K with varying pressures. As can be seen in Figure 5, the rapid switching behavior can be recovered for an external pressure of at least 5 GPa. This is in contrast to the zero-pressure modeling, where much larger temperatures are required to cause this behavior. Thus, the external pressure destabilizes the ferroelectric phase. Following the discussion of Íniguez and Vanderbilt [50], this can be explained by two counteracting effects. On the one hand, the lattice dynamical fluctuations, either thermal or quantum-mechanical in character, tend to favor the paraelectric cubic phase over the ferroelectric tetragonal phase. On the other hand, the potential-energy preference from first-principles calculations follows the reverse order [50]. At small pressures, the ferroelectric phase is more stable because the potential-energy contribution dominates. In contrast, as the system is compressed the potential-energy difference between the two phases decreases, which favors the quantum-mechanical fluctuations, evidenced here as rapid switching events, and thus the paraelectric phase. In comparison, to recover the frequent switching behavior at the chosen temperature of 150 K, an applied external pressure of 5 GPa is sufficient. (Figure 5c). As a second example, the influence of pressure is given for the critical temperature of 400 K. In Figure 5d, the dynamics of the ensemble are analyzed for an external pressure of 10 GPa. Following our expectations, the data indicate a cubic unpolar phase, as in the zero-pressure case. It has to be noted that the rapid switching is even faster than for the zero-pressure case, with polarization states switching approximately each 100 fs. After subsequent analysis of the ensembles’ dynamics at the given temperature and pressure state variables, sampling the region of interest within the BaTiO_3_ phase diagram, it is now possible to predict the conditions at which the behavior of the statistical ensemble initiate the phase transition from the ferroelectric to the paraelectric phase. Thus, on this basis, part of the phase diagram in the vicinity of the phase boundary was assessed by means of ab initio molecular dynamics and compared to existing theoretical and experimental data (Figure 6).

For some AIMD calculations, it is not straightforward to decide if they represent a final cubic phase or initial tetragonal phase since they are in between both characteristics. These cases have been marked with semi-colored stars in the phase diagram. An example is given in Figure 5b. It represents the calculation at 100 K and 3 GPa. It shows a beginning transition, which stops and relaxes back to the tetragonal phase. The respective intermediate region in the phase diagram may be interpreted as phase transition hysteresis. As summarized in Figure 6, the presented data are in the best agreement with the experimental findings by Ishidate et al. [11]. In the comparison to Íniguez and Vanderbilt [50], our method of NpT-AIMD seems to be more realistic than the used Monte Carlo simulations. Their improvement with the path-integral quantum Monte Carlo (PI-QMC) technique can be reproduced by our calculations.

**Figure 7:**
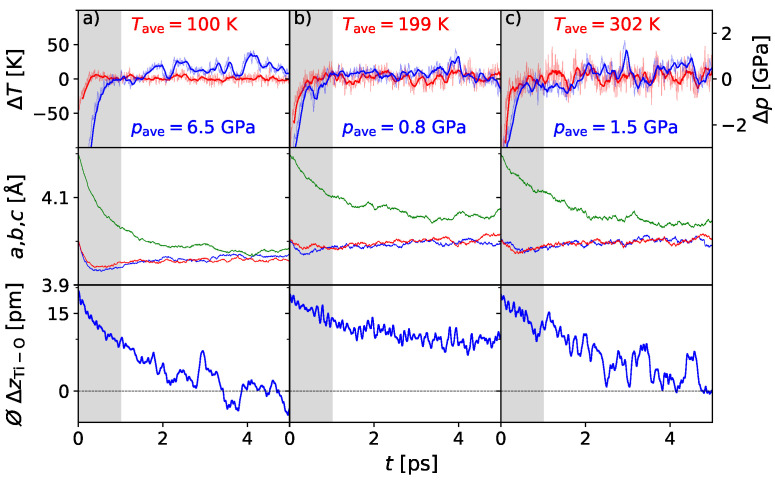
AIMD simulation of the BaTiO_3_ 4 × 4 × 4 supercell under the influence of chosen temperatures and external pressures: in (**a**) at 100 K and 7 GPa, (**b**) at 200 K and 1 GPa, and (**c**) at 300 K and 1 GPa. Lattice parameters *a, b,* and *c* given in red, blue and green, indicate the cubic phase for 100 K and 7 GPa. The mean displacement $⌀\Delta z_{\text{Ti-O}}$ reflects polar character for 200 K and 1 GPa, while for 300 K at 1 GPa the behavior represents the transition state. For the other conditions, centrosymmetry is observed.

To check the convergence with respect to the ensemble size and verify the identified temperature–pressure dependence of $T_{C}$, the influence of pressure has been modeled for selected state conditions (*p*, *T*) along the phase boundary within the 4 × 4 × 4 supercells with the determined Hubbard U. The results are presented in Figure 7. Here, we can see that the pressure effect is given correctly by the applied AIMD tracing again the experimental ferroelectric phase transition of barium titanate, joining with the already demonstrated zero-pressure isobar scenario under varying temperatures.

In comparison to previous work [33], the pressure-dependent behavior of the phase transition was successfully simulated using the barostat. These results presented in a phase diagram provide a good comparison with the literature.

## 4. Conclusions

In this work, supercells of barium titanate have been analyzed, reassessing the phase transition from the ferroelectric tetragonal phase to the paraelectric cubic phase by means of the AIMD method. Further calculations showed that the external pressure is able to suppress this switching. In the 2 × 2 × 2 supercell, strong fluctuations occur regarding temperature and pressure; in the 3 × 3 × 3 supercell, these fluctuations are suppressed. Here, a first switching of polarity is visible at 150 K. At a temperature of 400 K, the switching occurs rapidly, which is interpreted in average as the centrosymmetric paraelectric structure. The lattice parameters also indicate the cubic phase. Equivalent results can be achieved for a temperature of 150 K when applying a pressure of 5 GPa. The behavior at 400 K does not change qualitatively due to applied external pressure, but switching dynamics are significantly faster.

We conclude that the presented simulations provide a sound basis to derive the ferroelectric properties of BaTiO_3_ from the atomic displacements and average atomistic structure of the ensemble. To obtain reliable results reflecting the transition temperature T*_C_* as well as the pressure dependence of the phase boundary, a supercell of 3 × 3 × 3 is needed and large enough to find good consistency with experimental findings, as well as other theoretical approaches. We summarize our results in a temperature–pressure phase diagram in comparison to available data from the literature. We find a good agreement with the cited work, although the criteria for phase assignment and respective inaccuracies in the allocation of the characteristics required the definition of a transition region, in particular at lower temperatures and higher pressures.

While checking the convergence with respect to a larger system size, we found a strong dependence of the phase stability of the tetragonal ferroelectric and cubic paraelectric phases on the Hubbard U parameter. Details are presented in the Supplementary Section S3 and can also be found in the literature [47]. Cohen and Krakauer [19] explain the influence of the Hubbard U parameter on the phase transition in detail. Following this work, the ferroelectric phase is in particular stabilized by Ti-O hybridization. It is further discussed that by increasing the energy of Ti *3d*-states, the variational degree of freedom reduces and so the ferroelectric state may become energetically unstable. However, although the effect of Hubbard U stabilizing the phase transition of barium titanate at lower temperatures and/or pressures can be explained in this way, we cannot reason why the supercells act differently on this influence. A detailed analysis of this behavior is indeed interesting and will be the subject of further work.

Following our calculations, it can be concluded that the order–disorder model is more suitable for the given ferroelectric phase transition of BaTiO_3_ than the displacive model.

This work shall emphasize the possibility of controlling ferroelectric phenomena using external pressure. The first-principle results may guide experimental setups with variable temperature and pressure regimes to switch ferroelectricity in BaTiO_3_ bulk crystals or films, which in turn may lead to modern ferroelectric devices, such as piezoelectrically induced or patterned acoustic resonators, sensors, or optical filters.

## Data Availability

Data are contained within the article and Supplementary Materials.

## Supplementary Materials

The following supporting information can be downloaded at: https://www.mdpi.com/article/10.3390/ma17051023/s1, Figure S1: AIMD simulation of the BaTiO_3_ 2 × 2 × 4 supercell at 1000 K and 0 GPa external pressure: Lattice parameters *a*,*b*,*c* indicate the tetragonal phase. The mean displacement $⌀\Delta z_{\text{Ti-O}}$ reflects that the phase is polar.; Figure S2: AIMD simulation of the BaTiO_3_ 4 × 2 × 2 supercell for (200 & 1000) K at 0 GPa: Lattice parameters *a*,*b*,*c* indicate the orthorhombic phase for 200 K (on the left side), while for 1000 K (on the right side) cubic phase is approached. $⌀\Delta z_{\text{Ti-O}}$ reflects that the phase is polar in case of 200 K. In addition, the polarization attains a contribution in *x*-direction $⌀\Delta x_{\text{Ti-O}}$. For 1000 K the mean displacements in *a* and *c* direction reflect centrosymmetric behavior. The switching of the dipole direction occurs rapidly in both directions; Figure S3: AIMD simulation of the BaTiO_3_ 2 × 2 × 2 supercell under the influence of chosen temperatures and external pressures: In (a) at 700 K and 0.1 GPa, (b) at 800 K at 1 GPa, (c) at 800 K at 5 GPa, (d) at 2000 K and 5 GPa. Looking at the averaged pressures p_ave_ a strong deviation between these values and the aimed values appear. Further, the pressure is fluctuating strongly in this supercell. For this reason, no interpretation of pressure effects is possible within the given supercell; Figure S4: Dependence of the total energy difference between the tetragonal and the cubic phase of barium titanate in relation to the Hubbard U parameter set to the Ti *d*-states.
