# Supplementary material for: Ferroelectric Phase Transition in Barium Titanate Revisited with Ab Initio Molecular Dynamics
Source: Materials (Basel). 2024 Feb 23;17(5):1023. doi: 10.3390/ma17051023 (PMC10934432; doi:10.3390/ma17051023)
Supplement: Supplementary file 1 [file materials-17-01023-s001.zip › materials-2866418-supplementary.pdf]

Supplementary Materials: Ferroelectric phase transition in barium titanate revisited by *ab initio* Molecular Dynamics

Christian Ludt [1,2,*], Dirk C. Meyer [1,2] and Matthias Zschornak [1,2,3]

S1. 2x2x4 and 4x2x2 supercells

Further, we tested the two unsymmetric supercells of size 2x2x4 and 4x2x2 with initial polarization along as well as perpendicular to the elongated dimension. For the case of the 2x2x4 supercell, no qualitative change of polarity is visible in the graphs even at high temperatures. In Figure S1 the results of the supercell at 1000 K are presented.

For the 4x2x2 supercell, a different behavior appears, as it can be seen in Figure S2. At low temperature, the polar axis has contributions in *a* as well as in *c* direction. The lattice parameters indicate an orthorhombic phase.

At elevated temperature, the dipoles change direction rapidly (Figure S2). Apparently, the difference occurring in between both unsymmetric supercells originates from the initial dipole alignment. Here, polarization is additionally stabilized in the long direction. The increased degrees of freedom given in the elongated ensemble arrangement are used to introduce polarization in terms of the order-disorder mechanism. Further, we admit more tests on Hubbard U parameter are needed to fit this important parameter to the experimental findings.

S2. Pressure influence in the 2x2x2 supercell

In Figure S3 the results are presented for 700 K at 0.01 GPa, 800 K at 1 GPa, 800K K at 5 GPa as well as 2000 K at 5 GPa. As discussed above, strong fluctuations appear not only in temperature but even stronger in pressure. Furthermore, the average pressure does not fit to the aimed pressure of the barostat. The size of the supercell is incapable of representing thermodynamic equilibrium, as the statistical data is insufficient. For this reason, further interpretations on the pressure effects in this supercell (2x2x2) are not reliable.

S3. Energy difference between cubic and tetragonal phase in dependence of the Hubbard U parameter

While we were trying to verify our identified temperature-pressure dependence of T_C for larger supercells, we found stability exclusively for the cubic phase when using the Hubbard U value of 4 eV for titanium *d*-states, as reported in literature. Although performing well for the 3x3x3 supercells, by testing different convergence parameters, we found that the Hubbard U value strongly affects the ground-state total energy difference of both phases and thus determines the stable phase at the given conditions. This influence on stability between tetragonal and cubic phase can be seen in Figure S4.

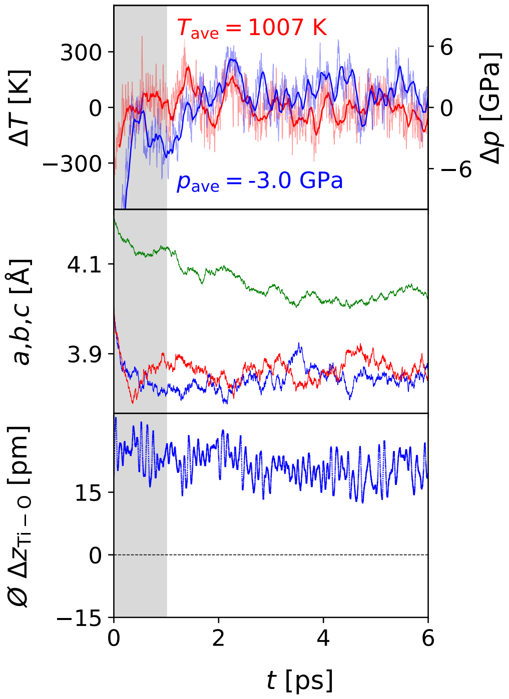

Figure S1. AIMD simulation of the $BaTiO_3$ 2x2x4 supercell at 1000 K and 0 GPa external pressure: Lattice parameters *a*,*b*,*c* indicate the tetragonal phase. The mean displacement $\varnothing\Delta z_{\text{Ti-O}}$ reflects that the phase is polar.

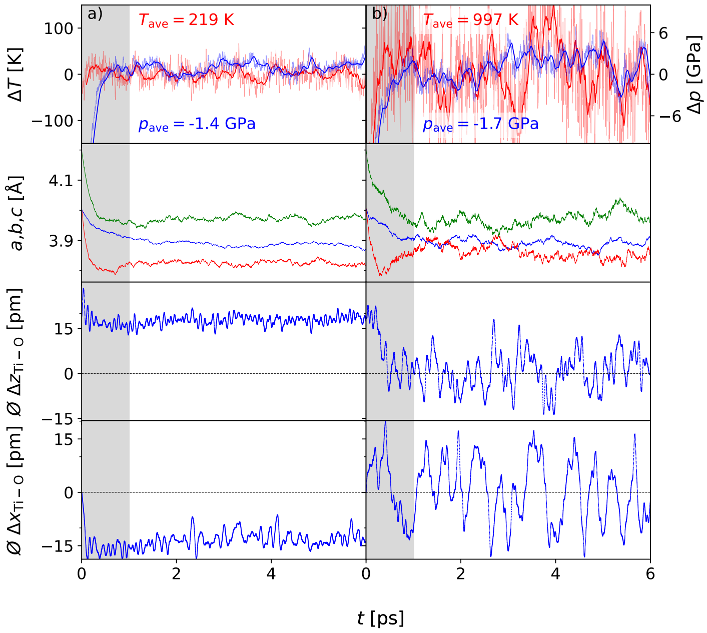

Figure S2. AIMD simulation of the $BaTiO_3$ 4x2x2 supercell for (200 & 1000) K at 0 GPa: Lattice parameters *a*,*b*,*c* indicate the orthorhombic phase for 200 K (on the left side), while for 1000 K (on the right side) cubic phase is approached. $\varnothing\Delta z_{\text{Ti-O}}$ reflects that the phase is polar in case of 200 K. In addition, the polarization attains a contribution in *x*-direction $\varnothing\Delta x_{\text{Ti-O}}$. For 1000 K the mean displacements in *a* and *c* direction reflect centrosymmetric behavior. The switching of the dipole direction occurs rapidly in both directions.

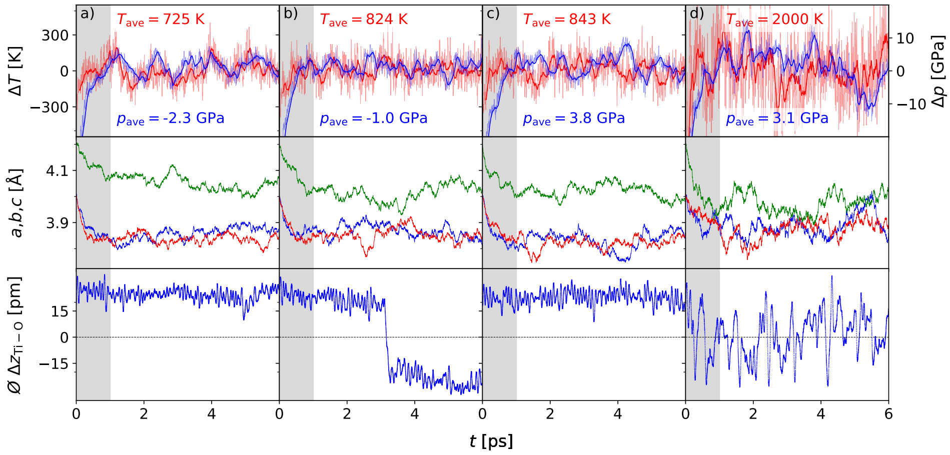

Figure S3. AIMD simulation of the $BaTiO_3$ 2x2x2 supercell under the influence of chosen temperatures and external pressures: In a) at 700 K and 0.1 GPa, b) at 800 K at 1 GPa, c) at 800 K at 5 GPa, d) at 2000 K and 5 GPa. Looking at the averaged pressures p_{ave} a strong deviation between these values and the aimed values appear. Further, the pressure is fluctuating strongly in this supercell. For this reason, no interpretation of pressure effects is possible within the given supercell.

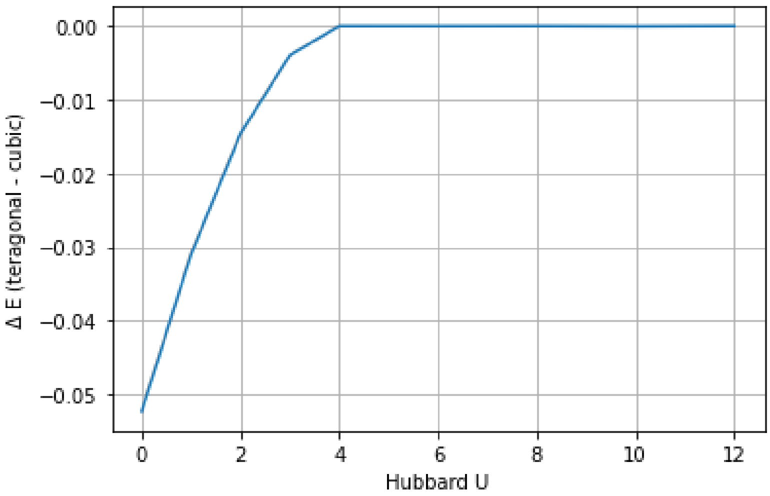

Figure S4. Dependence of the total energy difference between the tetragonal and the cubic phase of barium titanate in relation to the Hubbard U parameter set to the Ti *d*-states.
